# A Novel Heterozygous Mutation of the CYP17A1 Gene in a Child with a Micropenis and Isolated 17,20-Lyase Deficiency

**DOI:** 10.3390/ijerph19116880

**Published:** 2022-06-04

**Authors:** Maria Alessandra Saltarelli, Rossella Ferrante, Francesca Di Marcello, Daniela David, Silvia Valentinuzzi, Lucrezia Pilenzi, Luca Federici, Claudia Rossi, Liborio Stuppia, Stefano Tumini

**Affiliations:** 1 Department of Maternal and Child Health, UOSD Regional Center of Pediatric Diabetology, Chieti Hospital, 66100 Chieti, Italy; mariaalessandra.saltarelli@gmail.com (M.A.S.); francescadimarcello@gmail.com (F.D.M.); danieladavid88@gmail.com (D.D.); stefano.tumini@gmail.com (S.T.); 2 Department of Psychological, Health and Territory Sciences, School of Medicine and Health Sciences, “G. d’Annunzio” University of Chieti-Pescara, 66100 Chieti, Italy; lucrezia.pilenzi@studenti.unich.it (L.P.); claudia.rossi@unich.it (C.R.); stuppia@unich.it (L.S.); 3 Center for Advanced Studies and Technology (CAST), “G. d’Annunzio” University of Chieti-Pescara, 66100 Chieti, Italy; silvia.valentinuzzi@unich.it (S.V.); lfederici@unich.it (L.F.); 4 Department of Pharmacy, “G. d’Annunzio” University of Chieti-Pescara, 66100 Chieti, Italy; 5 Department of Innovative Technologies in Medicine and Dentistry, “G. d’Annunzio” University of Chieti-Pescara, 66100 Chieti, Italy

**Keywords:** 17,20-lyase deficiency, micropenis, CYP17A1 gene, heterozygous mutation, next-generation sequencing, mass spectrometry, steroid profiling

## Abstract

Disorders of sexual development (DSDs) are characterized by a heterogeneous group of congenital conditions associated with atypical development of the sex chromosomes, gonadal or anatomical sex. We report the case of a child with an isolated micropenis, a typical feature of the 46,XY DSD showing low basal testosterone levels and post-stimulation with the hCG test. Molecular analysis using a next-generation sequencing (NGS) panel of 50 genes involved in DSDs was performed, revealing a heterozygous mutation, c.1040G > ANM_000102.4, in the CYP17A1 gene. Sanger sequencing was used to confirm the gene variant detected by NGS; it was also performed to his parents, revealing the presence of the same mutation in the mother, who presented no features of the disease. Then, the serum steroid profile was determined by liquid chromatography coupled to tandem mass spectrometry analysis. Interestingly, this analysis highlighted low levels of testosterone, progesterone, and dehydroepiandrostenedione, as also confirmed by a stimulus test with ACTH. These results suggest that, in some cases, heterozygous mutations in recessive genes involved in adrenal steroidogenesis can also affect the patient’s phenotype.

## 1. Introduction

According to the International Consensus Conference on Intersex (Chicago 2006), micropenis, i.e., a penis with a stretched length of less than 2–2.5 standard deviation (SD) for age, can represent a clinical sign of various forms of disorders of sexual development (DSDs) [1,2]. In particular, in the context of 46,XY DSDs, micropenis is present in disorders of androgen synthesis, including congenital adrenal hyperplasia (CAH) and early androgen biosynthesis defects (for example, mutations and/or deficiencies in StAR, P450(scc), 3β-HSD II, P450R, and CYP17A1) [3]. The inclusion of CAH in newborn screening (NBS) programs has always been widely discussed internationally. The major reservations not to include CAH in NBS programs are related to the high false positive rates generated from immunoassay in low-birth-weight premature infants [4]. Therefore, several strategies have been applied worldwide to implement NBS programs to improve the specificity of the CAH screening test, thus also reducing the high false positive rates usually associated with the first-level CAH screening test by immunoassay. In particular, NBS laboratories worldwide have adopted the use of second-tier steroid profiling by liquid chromatography coupled to tandem mass spectrometry (LC–MS/MS) on a dried blood spot sample [5,6], the same specimen used for the first-level test, before the biochemical confirmation by serum steroid profiling by LC–MS/MS [7], and the final confirmation by molecular analysis. In fact, genetic testing plays a crucial role in the diagnosis of DSDs because the identification of the genetic basis of the disorder allows for early medical and therapeutic intervention. We report the case of a child with an isolated micropenis with specific basal and post-stimulus alterations of the adrenal steroid profile compatible with isolated 17,20-lyase deficiency. Indeed, a heterozygous CYP17A1 mutation was detected by a next-generation sequencing (NGS) panel designed in our laboratory and containing 50 genes involved in DSDs.

## 2. Materials and Methods

### 2.1. Case Report

A 5-year-old child was referred to the Regional Center of Pediatric Diabetology and Endocrinology of Chieti for expert consultation about micropenis.

The family history was unremarkable; the patient had two sisters in good general condition, and his parents were not consanguineous. He was born at full term after a normal pregnancy; the neonatal period was physiological. At two years of age, he was diagnosed with an autism spectrum disorder (ASD). No other major pathologies were reported. On physical examination, he was 120 cm tall and weighed 40 kg; the body mass index (BMI) was 27.8 kg/m^2^. He presented prepuberally with no pubic or axillary hair and prepubertal Tanner stage (P1G1). On clinical evaluation, the genitalia appeared phenotypically male, and the testicles were palpable bilaterally in the scrotum. He presented with a buried penis due to the presence of adiposity. However, proceeding with the stretched penis measurement with a rigid ruler, the length of the penis turned out to be about 3.5 cm, which led us to continue investigations. Specific details of the physical examination and clinical evaluation are reported below.

### 2.2. Physical and Clinical Evaluation

Body weight was determined to the nearest 0.1 kg using a weight scale, and height was measured to the nearest 0.1 cm using a Harpenden stadiometer. BMI was calculated as the ratio between the weight in kilograms and the height in meters squared (kg/m^2^). Puberal evaluation was assessed following Tanner staging. The stretched penile length (SPL) was measured in mm between the tip of the glans and the pubic symphysis, placing a ruler along the dorsum of the penis, pressing suprapubic fat tissue and fully stretching the penis. Blood pressure was evaluated using a calibrated sphygmomanometer. Three measurements were performed at 5 min intervals on the right arm after a 10 min rest in the supine position and then averaged. The cuff size was appropriate and equaled 40% of the arm circumference midway between the olecranon and the acromion according to the recommendation of the Task Force on Blood Pressure Control in Children [8]. Ambulatory blood pressure monitoring (ABPM) was used for the evaluation of the patient’s blood pressure. During a routine life day, 24 h monitoring was conducted. The assessment was adjusted for age, sex, body size, race, and ethnicity [9].

The first-level laboratory tests revealed normal electrolytes, renal, liver, bone, glucose, and thyroid profiles. The blood levels of gonadotropins, 17-beta estradiol, and adrenocorticotropic hormone (ACTH) were normal.

In order to rule out the presence of endocrine hypertension due to a possible deficiency of 17 hydroxylase/17,20-lyase, ambulatory blood pressure monitoring (ABPM) was also performed taking into consideration age, sex, body size, race, and ethnicity of the child.

### 2.3. Genetic Testing

Cytogenetic investigation was carried out with standard karyotype analysis. NGS analysis was carried out with a panel developed in our laboratory (Table 1).

Having obtained an informed consent form signed by both parents, genomic DNA was extracted from a buccal swab using a MagPurix instrument and a Forensic DNA Extraction Kit (Zinexts Life Science Corp., ZP01001) according to the manufacturer’s protocol.

NGS was performed using an Ion Torrent S5 system (Thermo Fisher, Applied Biosystems; Foster City, CA, USA) after library preparation consisting of fragmentation and adapter ligation onto the PCR products, clonal amplification. The sequencing step was carried out with Ion Torrent-specific equipment and reagents according to the manufacturer’s protocols. The prepared samples of ion sphere particles (ISP) were loaded onto an Ion 510™ chip with the Ion Chef (Thermo Fisher, Applied Biosystems; Foster City, CA, USA), and sequencing was performed using Ion S5™ sequencing reagents (Thermo Fisher, Applied Biosystems; Foster City, CA, USA). Analysis of the NGS data was performed using the software Ion Reporter 5.12 (Thermo Fisher; Foster City, CA, USA).

The uniformity of base coverage was over 98% in all the batches, and the base coverage was over 20× in all the target regions.

Sanger sequencing was used for the confirmation of gene variants detected by NGS. The same Sanger sequencing was performed for the unaffected parents of the child.

Then, multiplex ligation-dependent probe amplification (MLPA) analysis was carried out using the SALSA MLPA PROBEMIX P334 GONADAL (MRC Holland, Amsterdam, The Netherlands) containing 60 probes specific for four genes (DMRT1, CYP17A1, SRD5A2, and HSD17B3) which are simultaneously used in a single reaction.

### 2.4. Serum Steroid Profiling by Liquid Chromatography Coupled to Tandem Mass Spectrometry

Following the results obtained with the genetic test, serum steroid profiling was also determined by performing LC–MS/MS analysis using an in vitro diagnostic reagent kit, CHS™ MSMS Steroids Kit (PerkinElmer^®^, Turku, Finland) as already described [10,11,12]. The steroid profiling involved the measurement of cortisol (CORT), corticosterone (CCONE), 11-deoxycortisol (11-DECOL), dehydroepiandrostenedione (DHEA), dehydroepiandrostenedione sulfate (DHEAS), 4-androstene-3,17-dione (ADIONE), testosterone (TESTO), 17α-hydroxyprogesterone (17-OHP), and progesterone (PROG).

### 2.5. Stimulation Tests

In addition to the basal hormone dosage, a testicular stimulation test with human chorionic gonadotropin (hCG) was performed by intramuscular administration of 1000 IU of hCG per day for three consecutive days. A blood sample was taken on the first day (T0), before the first administration, and on the fourth day (T1) to evaluate the testosterone, dihydrotestosterone, and DHEA levels before and after stimulation.

Following the NGS response, in order to better characterize the peripheral metabolism of testosterone and based on the data present in the literature, a stimulus test with ACTH was performed. More specifically, stimulation with ACTH was performed by intravenous administration of 0.25 mg of ACTH. Blood samples were collected at Time 0 (T0′) and 60 min after the stimulus (T1′) for serum steroid profile determination by LC–MS/MS at the basal and post-stimulation timepoints.

## 3. Results

The first basal serum steroid profile and hormonal values are described in Table 2. The LC–MS/MS analysis revealed very low levels of testosterone, progesterone, and dehydroepiandrosterone (DHEA). In fact, these steroids were not measurable because their concentration levels were under the limit of quantification of the LC–MS/MS analytical method (normal values (n.v.), respectively, 0.03–9.7 ng/mL, 0.07–12.94 ng/mL, and 0.09–19 ng/mL), while the glucocorticoid and mineralcorticoid profiles were within the normal range).

Testicular stimulation with hCG (Table 3) showed no increase in testosterone and androstenedione levels, with a very low increase in dihydrotestosterone levels, which was below the sensitivity level of the method. The result of the hCG stimulus test associated with an insufficient response of dihydrotestosterone on the fourth day after three administrations of hCG led us to suspecting a deficit of peripheral metabolism of testosterone.

**Figure 1:**
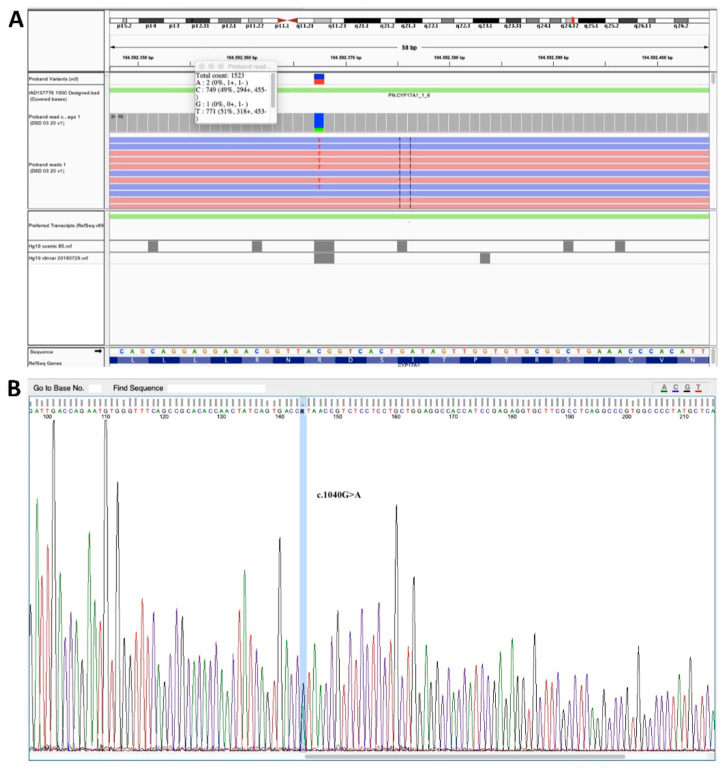
NGS analysis and Sanger sequencing of exon 6 of CYP17A1. (**A**) Heterozygous c.1040G > A (p.Arg347His) mutation in the CYP17A1 gene as highlighted by NGS analysis. (**B**) Sanger sequencing of exon 6 of CYP17A1.

According to the cytogenetic investigation, the patient showed a normal 46,XY karyotype. NGS analysis evidenced the presence of a heterozygous c.1040G > ANM_000102.4 (p.Arg347His) mutation in the CYP17A1 gene which was confirmed by Sanger sequencing of exon 6 of this gene (Figure 1). Segregation analysis in the unaffected parents showed the presence of the same mutation in the mother of the child, who, however, presented no features of the disease. No large deletions of exons were found by MLPA analysis.

The profile of the basal and post-ACTH stimulus serum steroids is showed in Table 4. There was no response of testosterone, associated with a very low increase in androstenedione (T0′ = 0.15 ng/mL, T1′ = 0.20 ng/mL, n.v.: 0.06–2.6 ng/mL) and dehydroepiandrosterone (T0′ = 0 ng/mL, T1′ = 0.25 ng/mL, n.v.: 0.09–19 ng/mL). In particular, this test showed a marked increase in 17-OH progesterone (T0′ = 0.30 ng/mL, T1′ = 4.85 ng/mL, n.v.: 0.03–2.65 ng/mL), and 11-deoxycortisol (T0′ = 0.25 ng/mL, T1′ = 1.35 ng/mL, n.v.: 0.1–1.56 ng/mL). These findings, combined with normal blood pressure values and normal potassium levels, suggested a prevalent impairment of the 17,20-lyase activity. A significant response to the stimulus was also highlighted for cortisol and corticosterone.

## 4. Discussion

In the present study, we described the clinical evaluation, genetic diagnosis, and molecular mechanisms underlying the finding of a micropenis in a child with a normal male karyotype. There are several possible causes of micropenis, including hypogonadotropic hypogonadism, testicular dysgenesis, testosterone synthesis alterations, androgen resistance caused by 5a-reductase deficiency or partial androgen insensitivity and other rare causes. In many cases, the underlying etiology remains unknown, characterizing the so-called isolated idiopathic micropenis [2].

**Figure 2:**
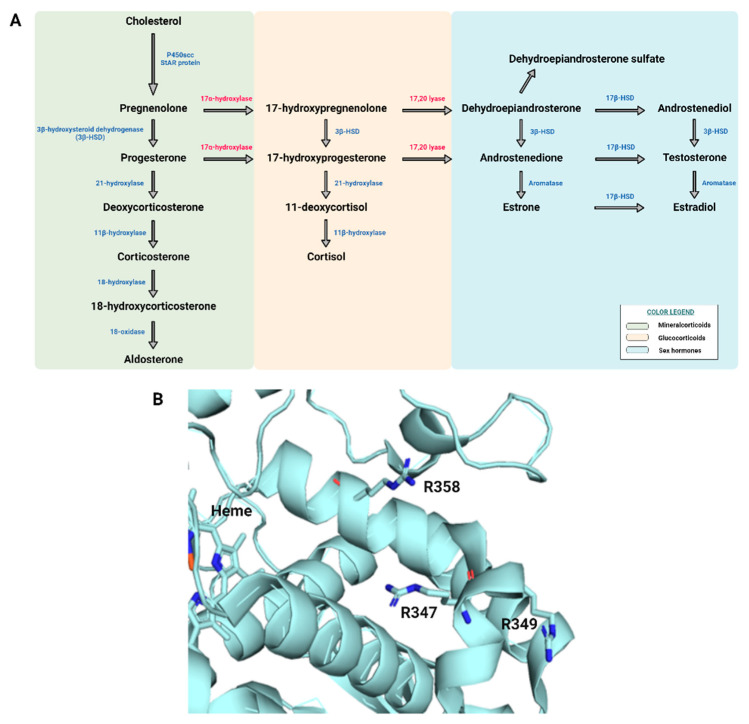
Steroidogenesis and human CYP45017A structure. (**A**) Schematic representation of the adrenal steroidogenesis biosynthesis pathway. (**B**) Ribbon representation of the human p450c17 structure. R347 is located on the proximal face of the enzyme with respect to the heme group. The position of residues R349 and R358 is also highlighted. All three residues have been found mutated in CAH patients and their mutations affect the lyase activity only, consistent with their implication in the interaction with cytochrome b5 (PDB code 3RUK).

In this study, a heterozygous c.1040G > A (p.Arg347His) mutation was detected in the CYP17A1 gene by NGS analysis. This gene is located on chromosome 10q24.3 and produces an identical mRNA both in the adrenals and the gonads in healthy subjects. The CYP17A1 gene encodes the p450c17 enzyme which catalyzes steroid 17α-hydroxylase and 17,20-lyase activities [13]. The 17α-hydroxylase/17,20-lyase deficiency caused by a mutation in the CYP17A1 gene is the least common form of CAH, accounting for less than1% of all cases [14]. Figure 2 panel A represents the adrenal steroidogenesis biosynthesis pathway, with the two enzyme’s activities, 17α-hydroxylase and 17,20-lyase, reported in red. Moreover, a ribbon representation of the human p450c17 structure is shown in Figure 2 panel B, with the position of the three residues R347, R349, and R358 highlighted with sticks. In particular, all three residues have been found mutated in CAH patients and their mutations affect the lyase activity only, consistent with taking part in the interaction surface with cytochrome b5.

Importantly, 17,20-lyase deficiency is reported to be less frequent than 17-hydroxylase deficiency [15], and it is usually difficult to distinguish between these forms in terms of the clinical and biochemical outcomes [16]. The two enzyme’s activities are responsible for the synthesis of 17OH-pregnenolone from pregnenolone and of 17OH-progesterone from progesterone and the production of DHEA from 17OH-pregnenolone [17]. Therefore, CYP17A1 is the exclusive gateway to sex steroid production. Approximately 129 different *CYP17A1* gene variants have been reported, including missense variants, small insertions or deletions, and splice site variants mostly associated with complete loss of both 17-hydroxylase and 17,20-lyase activities; however, partial loss of both enzymatic activities and loss of either hydroxylase or lyase activity alone have also been reported [13]. Our patient’s mutation was previously reported in a male patient suffering from genital ambiguity, who, however, presented a homozygous mutation at this site, as well as in other patients with compound heterozygous mutations of CYP17A1 [18,19]. As already reported in the literature for other autosomal recessive diseases, a single variant in heterozygosity may be associated with a disease phenotype, as in the cases of familial Mediterranean fever (FMF) with heterozygous MEFV developing periodic fever [20,21,22]. In the present case report, segregation analysis of the parents of the child revealed the presence of the heterozygous mutation in the mother only, who never presented any symptoms of the disease.

The absence of basal testosterone and its precursors, confirmed by hCG and ACTH tests, associated with a response of the glucocorticoids and mineralocorticoids profile to the ACTH stimulus test led us to hypothesizing a reduction in the 17,20-lyase activity of p450c17 not affecting the hydroxylase activity. This hypothesis was also clinically confirmed by the absence of some typical features of the combined deficiency of 17-hydroxylase/17,20-lyase activities, such as hypokalemia and hypertension. Final confirmation came from molecular analysis which allowed the identification of the heterozygous c.1040G > A variant leading to an Arf347His mutation. Indeed, it has already been shown that the mutation of Arg347 causes a deficient interaction between cytochrome b_5_ and CYP17, with the subsequent reduction of 17,20-lyase activity only [23].

The segregation analysis of the parents showed that the c.1040G > A variant was maternally inherited. The presence of the heterozygous mutation in the mother who does not have the disease is not in contrast with the evidence found.

Similar features were already described in some cases with homozygous or combined heterozygous mutations [18,24]. In particular, Biason-Lauber et al. described the case of a newborn patient with a normal 46,XY karyotype and a compound heterozygous mutation of the CYP17A1 gene who presented a micropenis, with undetectable basal hormone values, normal responses of 17-hydroxyprogesterone and cortisol to ACTH, and no androgen increase after hCG stimulation [24].

In addition, our patient was diagnosed with an ASD. The association between the alteration of steroidogenesis and ASDs is still debated. Some theories, such as “the extreme male brain theory” or “the fetal androgen theory,” find the cause of ASDs in prenatal exposure to male steroid hormones, especially testosterone [25]. Similarly, studies about postnatal androgen levels in ASDs are still controversial [26], but it has been hypothesized that ASD children may present upregulated CYP17A1 activity [27]. No correlation between low androgen levels and ASDs has been described yet.

To the best of our knowledge, this is the first report describing a 17,20-lyase deficiency caused by a heterozygous mutation of the CYP17A1 gene. The presence of a mutation in heterozygosity suggests that it could probably allow sufficient steroidogenesis to maintain normal sex differentiation. However, the presence of low levels of TESTO, PROG, and DHEA suggests that probably in this case the heterozygous mutation in the gene could have caused some symptoms of the disease.

As is well-recognized, NGS is a high-throughput methodology that enables rapid sequencing of the base pairs in DNA samples. Importantly, NGS drives discovery of new or rare mutations in a short time compared to classical molecular biology techniques, such as direct sequencing, thus supporting molecular analysis and a broad range of applications. It is worth noting how in the present case the application of a specific NGS panel made it possible to analyze simultaneously several genes involved in the DSD. In particular, the specific gene panel that we designed was developed to screen clinical variants related to DSDs. Of interest, screening with the NGS panel allowed identifying the genetic etiology of some patients with DSDs, providing a precision science basis for clinical phenotype classification and genetic counseling.

## 5. Conclusions

This case report suggests that, in some cases, heterozygous mutations in the genes involved in adrenal steroidogenesis may cause a pathological phenotype despite the presence of a wild-type allele.

## Figures and Tables

**Table 1:** NGS panel containing 50 genes involved in DSDs.

	*Gene*	*OMIM*	*Refseq*		*Gene*	*OMIM*	*Refseq*
1	AKR1C2	600450	NM_001354.5	26	HSD17B4	601860	NM_000414.3
2	AKR1C4	600451	NM_001818.3	27	HSD3B2	613890	NM_000198.3
3	ANOS1	300836	NM_000216.3	28	INSL3	146738	NM_005543.4
4	AR	313700	NM_000044.3	29	LEP	164160	NM_000230.2
5	ATRX	300032	NM_000489.3	30	LHCGR	152790	NM_000233.3
6	AMHR2	600956	NM_020547.3	31	MAMLD1	300120	NM_005491.3
7	BMP15	300247	NM_005448.2	32	MAP3K1	600982	NM_005921.2
8	CHD7	608892	NM_017780.3	33	NR0B1	300473	NM_000475.4
9	DMRT1	601898	NM_004122.2	34	NR3C1	138040	NM_001018077.1
10	CYB5A	613218	NM_001914.3	35	NR5A1	184757	NM_004959.4
11	CYP11A1	118485	NM_000781.3	36	POR	124015	NM_000941.2
12	CYP11B1	610613	NM_000497.4	37	PROK2	607002	NM_00112128.1
13	CYP17A1	609300	NM_000102.4	38	PROKR2	607623	NM_144773.3
14	CYP19A1	107910	NM_000103.3	39	RXFP2	606655	NM_130806.3
15	DHH	605423	NM_021044	40	SOX9	608160	NM_000346.3
16	FGF8	600483	NM_006119.4	41	SRD5A2	607306	NM_000348.3
17	FGFR1	136350	NM_023110.2	42	WDR11	606417	NM_018117.11
18	FGFR2	176943	NM_000141.4	43	SRY	480000	NM_003140.3
19	FSHB	136530	NM_000510.2	44	WT1	607102	NM_024426.4
20	FSHR	136435	NM_000145.4	45	STAR	600612	NM_000349.2
21	GATA4	600576	NM_002052.3	46	TAC3	162330	NM_001178054.1
22	GNRH1	152760	NM_001083111.1	47	ZFPM2	603693	NM_012082.3
23	GNRHR	138850	NM_000406.2	48	PROP1	601538	NM_006261.4
24	HESX1	601802	NM_003865.2	49	RSPO1	609595	NM_001038633.3
25	HSD17B3	605573	NM_000197.1	50	DMRT2	602424	NM_021951.2

**Table 2:** Serum steroids and pituitary hormones at the time of presentation using different reporting units (compared with the recommended pediatric reference ranges).

Laboratory Test	Result	Reference Range
**FSH (IU/L)**	0.9	0.95–11.95
**LH (IU/L)**	0.0	0.57–12.07
**ACTH (ng/mL)**	12.7–38.1	4.7–48.8
**17 beta-Estradiol (pg/mL)**	<10	11–44
**Cortisol (ng/mL)**	75.8	10–330
**Corticosterone (ng/mL)**	2.4	0.8–18.6
**11-Deoxycortisol (ng/mL)**	0.4	0.1–1.56
**Androstenedione (ng/mL)**	0.1	0.06–2.6
**17α-Hydroxy-Progesterone (ng/mL)**	0.4	0.03–2.65
**Testosterone (ng/mL)**	/	0.03–9.7
**Progesterone (ng/mL)**	/	0.07–12.94
**DHEA (ng/mL)**	/	0.09–19
**DHEA-S (** **ng/mL)**	400	50−4420

**Table 3:** Serum steroid levels before and after stimulation with hCG.

Laboratory Test	T0 (before Stimulus)	T1 (4th Day after Stimulus)	Reference Range
**Testosterone (ng/mL)**	<0.2	<0.2	2.12–7.42
**Dihydrotestosterone (pg/mL)**	45	62	250–990
**DHEA (ng/mL)**	<0.3	<0.3	0.3–1.9

**Table 4:** Serum steroid levels measured before and after stimulation with ACTH.

Laboratory Test	T0’ (before Stimulus)	T1’ (60 min after Stimulus)	Reference Range
**Cortisol ng/mL**	43.85	207.85	10–330
**Corticosterone ng/mL**	1.20	39.15	0.8–18.6
**11-Deoxycortisol ng/mL**	0.25	1.35	0.1–1.56
**Androstenedione ng/mL**	0.15	0.20	0.06–2.6
**17α-Hydroxy-Progesterone ng/mL**	0.30	4.85	0.03–2.65
**Testosterone ng/mL**	/	/	0.03–9.7
**Progesterone ng/mL**	0.05	1.90	0.07–12.94
**DHEA ng/mL**	/	0.25	0.09–19
**DHEA-S** **ng/mL**	482.75	453.2	50–4420
